# Effects of Radiation Therapy on Neural Stem Cells

**DOI:** 10.3390/genes10090640

**Published:** 2019-08-24

**Authors:** Anna Michaelidesová, Jana Konířová, Petr Bartůněk, Martina Zíková

**Affiliations:** 1 Laboratory of Cell Differentiation, Institute of Molecular Genetics of the Czech Academy of Sciences, v. v. i., Vídeňská 1083, 142 20 Prague 4, Czech Republic; 2 Department of Radiation Dosimentry, Nuclear Physics Institute of the Czech Academy of Sciences, v. v. i., Na Truhlářce 39/64, 180 00 Prague 8, Czech Republic

**Keywords:** neural stem cells, brain and nervous system cancers, neurogenic niches, radiotherapy, sparing of neurogenic regions

## Abstract

Brain and nervous system cancers in children represent the second most common neoplasia after leukemia. Radiotherapy plays a significant role in cancer treatment; however, the use of such therapy is not without devastating side effects. The impact of radiation-induced damage to the brain is multifactorial, but the damage to neural stem cell populations seems to play a key role. The brain contains pools of regenerative neural stem cells that reside in specialized neurogenic niches and can generate new neurons. In this review, we describe the advances in radiotherapy techniques that protect neural stem cell compartments, and subsequently limit and prevent the occurrence and development of side effects. We also summarize the current knowledge about neural stem cells and the molecular mechanisms underlying changes in neural stem cell niches after brain radiotherapy. Strategies used to minimize radiation-related damages, as well as new challenges in the treatment of brain tumors are also discussed.

## 1. Introduction

During 2018, 17 million new cancer cases and 9.6 million cancer-associated deaths were reported worldwide [1]. According to the International Agency for Research on Cancer, the worldwide estimated incidence of brain and nervous system cancers in 2018 for both sexes and all ages was 3.5 per 100,000 population, being the 18th most common cancer site [2]. The incidence of brain and nervous system cancers for both sexes and ages from 0 to 19 years old was 1.2 per 100,000, making it the second most common cancer site after leukemia for this age group [2].

Primary brain tumors can be divided into several categories, such as tumors of neuroepithelial tissue (e.g., astrocytoma, glioblastoma), ependymal, choroid plexus, pineal parenchymal, embryonal (medulloblastoma), meningeal tumors, and primary central nervous system (CNS) lymphomas [3]. The most commonly diagnosed CNS tumors, occurring as much as 10 times more frequently than primary malignant brain tumors, are intracranial or brain metastases (BM) [4,5]. Brain metastases are mostly connected to lung, breast, colon, and skin (melanoma) primary cancers [4,5,6,7]; they occur in approximately 30% of all cancer patients [8], and most of the BM patients develop multiple intracranial BMs [9]. They are mostly located at the gray–white matter, with 80% occurrence in the cerebral hemispheres, 15% in the cerebellum, and 5% in the brainstem [9].

There are several options for cancer treatment including surgery, chemotherapy, immunotherapy, hormonotherapy, radiotherapy, and others. Selection of the most appropriate treatment strategy depends on several parameters, such as the cancer site/type and stage [10]. In general, radiotherapy seems to be an appropriate treatment in more than 50% of all cancer patients [11] and it is, next to surgery, the standard treatment strategy for most primary CNS malignancies and BMs [7,12].

Chemotherapeutic treatment of CNS tumors is hampered by the blood–brain barrier (BBB), which protects the brain from exposure to toxins, and, thus– blocks the entry of many water-soluble drugs from the blood into the brain parenchyma [7,13]. One of the most studied proteins that play a significant role in the BBB is efflux transporter permeability glycoprotein, also known as ATP-binding cassette sub-family B member 1 (ABCB1) [12]. It was shown that inhibition of this protein in in vivo models increases the brain penetration of several chemotherapeutic agents [14,15,16,17]. Unfortunately, clinical trials using permeability glycoprotein inhibitors showed unacceptable toxicities and were terminated early [18]. More recently, inhibition of a related protein, breast cancer resistance protein ABCG2, was found to increase the permeability of BBB in the mouse [19], thus identifying an alternative molecular target for potential adjuvant therapy. Radiotherapy can also disrupt the BBB, increasing the penetration of chemotherapeutic agents to the brain [13,20,21,22]. Due to this effect of radiotherapy, it is often beneficial to use a combination of radiotherapy and chemotherapy, known as chemoradiotherapy [23,24].

Brain radiotherapy improves the lives of cancer patients and concurrently, advances in these techniques allow a significant increase in the proportion of patient survivors. However, the use of these therapies is not without devastating side effects that impact the patients’ autonomy, as well as their social and professional life. Although the effect of radiation-induced damage to the brain is multifactorial, injury to the neural stem cell (NSC) compartments and damage to NSC populations is hypothesized to be central to the pathogenesis of radiation-induced cognitive decline. Sensitivity of NSC compartments to radiation has been extensively studied using rodent models, also permitting the study of possible links between cancer therapy and the onset of cognitive deficits.

## 2. Radiotherapy Techniques

### 2.1. Techniques for Delivering Radiation Therapy

The main aim of radiotherapy is to destroy cancer cells while causing minimal damage to the surrounding healthy tissues. Indeed, this is not always possible, and in some cases even not applicable, for example during total body or whole brain irradiation.

Radiotherapy can be divided into external and internal. In external radiotherapy, ionizing radiation is delivered to the patient’s body using external beams consisting of either photons, electrons, neutrons, protons, or other ions (e.g., carbons). Internal radiotherapy can be divided into brachytherapy and nuclear medicine. In brachytherapy, small sources of ionizing radiation are delivered inside or to the proximity of the tumor [25], and, in the case of nuclear medicine, radiopharmaceutical agents are delivered into the patient’s body using specialized molecular vehicles [26].

The therapeutic dose is mostly delivered to the patients in several doses (so-called fractionation). This means that the patient is not irradiated in one session, but the dose is delivered in parts. It was shown that the time needed for the repair of cancer cells is longer than in case of normal (healthy) cells. This means that by using multiple optimally spaced irradiation sessions, normal cells will have time to repair and the cancer cells will be preferentially eliminated [27]. Another factor that makes fractionation beneficial is the cell cycle dependency of cellular radiosensitivity. In an asynchronous cell population, cells in M phase will be more likely killed by radiation than cells in G_1_ or S phase. Thus, irradiating cancer cells in more than one session increases the probability of their elimination [28].

In general, irradiation limited to cancer cells only is impossible. The radiation is usually directed to a restricted body volume (defined by the physician), which is selected based on the tumor histology and location. Most used for external radiotherapy are photon beams (X-rays). These X-rays are generated inside a clinical linear accelerator (LINAC). A wide X-ray beam is then extracted from the LINAC for patient irradiations. The LINACs are able to rotate around the patient and are equipped with collimators that reduce the size of the photon beam to a square region that through an additional collimation system, mostly a Multi-leaf collimator (MLC), can be adjusted to copy the treatment volume shape.

In most cases, the treatment dose is not delivered from one direction only, but sophisticated treatment planning systems are used for the calculation of the most appropriate dose distributions. The treatment plan is always constructed based on the actual patient’s anatomy obtained mostly by computed tomography. Recently, a large percentage of treatment plans are prepared using the approach of intensity-modulated radiotherapy (IMRT), where the dose is delivered using non-uniform beams by the use of MLC from multiple directions. This approach enables the physician to achieve delivery of the full treatment dose only within the designated treatment volume, with maximal sparing of the healthy tissues [29].

### 2.2. Brain Radiotherapy

In case of brain tumors, the whole brain, or only parts of it, can be irradiated [30]. Whole brain radiation therapy (WBRT) has been routinely used since the 1960s in cases of multiple BMs [31,32]. As the incidence of BM in NSC regions was found to be low, sparing of the neurogenic compartments could help reducing the neurocognitive decline observed after WBRT [31]. The neurogenic niches can be spared using the above-mentioned IMRT techniques based on photons or alternatively, delivering protons using the pencil-beam scanning (PBS) mode [33]. In PBS, it is possible to irradiate the patient’s volume using a thin pencil beam, which is redirected using magnets to smaller sub-volumes of the total volume to facilitate more conformal irradiation while sparing healthy tissues.

Although shown to prolong a patient’s life, WBRT is also associated with several side effects such as hair loss, skin irritation, nausea, hearing loss, cerebral edema, radionecrosis, neurological deterioration, cognitive and endocrine dysfunctions, and dementia [32,34]. Less side effects were observed when using stereotactic radiosurgery (SR). During SR, a high dose is delivered using multiple focused beams to the brain regions where metastases are located. This can be achieved by the use of modified LINACs, i.e., use of stereotactic tubes or microMLC in order to restrict the beam size to a smaller area, multiple ^6^°Co sources from several directions (Gammaknife), or robotic LINAC (Cyberknife). Stereotactic radiosurgery is in general less invasive and is mostly executed in one session due to the possibility to irradiate small tissue volumes and minimally affect the healthy tissues [32]. In many cases, SR can be used instead of surgery in combination with WBRT [35], or as an adjuvant therapeutic strategy after resection of the metastases [8]. In addition to these external beam techniques, radioactive sources can be implanted into the tumor cavity during surgery (intracranial brachytherapy) [8,25]. These radioactive implants can be placed into the patient permanently or temporarily. As temporary implants necessitate an additional surgery, permanent implants are more preferred [8].

In external radiotherapy and brachytherapy, cancer cells cannot be irradiated selectively, but always a targeted volume is irradiated, which contains healthy cells as well. However, nuclear medicine offers the possibility to treat cancer using targeted radiotherapy. During targeted radiotherapy, molecular vehicles are used to selectively deliver a radionuclide to malignant cell populations [26]. For example, glioblastoma multiforme cells highly express G protein-coupled receptor neurokinin 1, so a modified substance P as its ligand (^213^Bi-DOTA-Substance P, where ^213^Bi is a short-range alpha particle emitter) can be used for targeting neurokinin type 1 receptor-producing cells [36]. The used radionuclides mostly emit electrons with a range of a few millimeters, or they can emit alpha particles with a range of only a few cell diameters [26]. The low range of these beta and alpha emitters, respectively, reduces irradiation of the healthy tissues and thus the unwanted side effects of radiotherapy.

### 2.3. Side Effects of Radiation Therapy

The unwanted side effects of radiation therapy can be divided into three categories: acute, subacute, and late [37]. Acute effects are mostly caused by BBB disruption leading to cerebral edema, and they may be improved using corticosteroid medications [38,39,40]. These effects occur during the first few weeks of radiotherapy and are characterized by drowsiness, headache, fever, nausea, and vomiting [39,40]. Subacute effects occur one to six months post-irradiation, and they include several symptoms such as headache, somnolence, weakness, anorexia, and aggravation of preexisting deficits [39,40]. Late effects are mostly irreversible, and they appear more than six months after the treatment and are associated with white matter damage caused by vascular injuries, demyelination, or radiation-induced necrosis [39,40,41]. These effects can be mild, such as tiredness, or significant, such as memory loss, dementia [39], leukoencephalopathy [42], and secondary-induced brain tumors (meningioma, glioma, sarcoma) [40]. Importantly, late effects are even more severe for pediatric patients; childhood cancer survivors are increasingly predisposed to cognitive deficits [33,43]. It was observed that long-term survivors of brain cancer irradiations in childhood suffer losses in intelligence quotient, learning disabilities, hormonal deficits, growth and psychomotor retardation [38]. Some of these pathological states are associated with the radiation damage to the neurogenic niche, which is involved in memory formation, spatial processing, and mood regulation [44].

## 3. Neural Stem Cells

The adult brain has long been considered limited in its regenerative capacity; it was believed that neurogenesis ceased after development. However, over 50 years ago, this concept was changed after neurogenesis in the adult mammalian brain was discovered [45]. Since then, enormous progress has been made in the understanding of this process. Neural stem cells are undifferentiated cells that are defined by their replicative potential and their ability to differentiate into multiple neuronal and glial cell types, as well as their capacity for long-term self-renewal. The adult brain contains two NSC pools located in the sub-ventricular zone of the lateral ventricles (V-SVZ) [46] and the dentate gyrus of the hippocampus [47]. Both NSC pools produce new neurons that can integrate into functional circuits [48,49]. Although high proliferative capacity is a feature of the ‘stemness”, another unique characteristic of NSCs is their ability to stay dormant for long periods, providing a reserve pool of cells available for tissue regeneration throughout life [50]. As radiotherapy exerts its effect on dividing cells, leading them to stop proliferation, the cognitive decline in patients indicates a dysfunction in mitotically active NSCs.

Most of the findings on NSC behavior derive from studies in rodent models, and the knowledge about NSCs in human brain is still very limited. Whether neurogenesis in humans exists has been investigated using various approaches, such as BrdU incorporation [51] and carbon dating [52], and has brought conclusive evidence about the presence of adult neurogenesis in the human brain. However, two recently published reports with opposite conclusions have reopened discussion concerning the existence of human adult hippocampal neurogenesis. Sorrells et al. [53] reported that there is no evidence of hippocampal neurogenesis in humans after adolescence, while Boldrini et al. [54] demonstrated the opposite by showing that adult neurogenesis persists during life, although with a small decrease with aging. Comparative analyses of adult neurogenesis have uncovered a large variance in this phenomenon among different species [55]. The neurogenesis in the V-SZV niche differs between humans and mice, based on the cell types that form this area [56]. Also, newly formed neural progenitors in this zone have distinct fates, becoming medium spiny neurons in human striatum [57], instead of forming olfactory interneurons as in mice [58].

**Figure 1:**
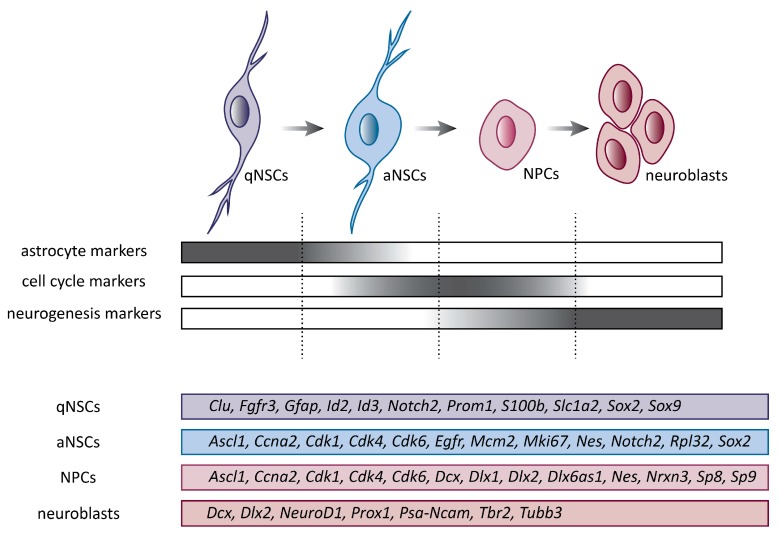
Cell subtypes involved in progression from quiescent neural stem cells (qNSCs) to neuroblasts. Schematic representation of lineage progression. QNSCs give rise to activated neural stem cells (aNSCs), which differentiate into highly proliferative progenitor cells (NPCs) and finally to neuroblasts. Expression of key genes related to particular cell subtypes is depicted. *Ascl1*, achaete-scute family bHLH transcription factor 1; *Ccna2*, cyclin A2; *Cdk1*, cyclin dependent kinase 1; *Cdk4*, cyclin dependent kinase 4; *Cdk6*, cyclin dependent kinase 6; *Clu*, clusterin; Dcx, doublecortin; *Dlx1*, distal-less homeobox 1; *Dlx2*, distal-less homeobox 2; *Dlx6as1*, distal-less homeobox 6, opposite strand 1; *Egfr*, epidermal growth factor receptor; *Gfap*, glial fibrillary acidic protein; *Id2*, inhibitor of DNA binding 2; *Id3*, inhibitor of DNA binding 3; *Mcm2*, minichromosome maintenance complex component 2; *Mki67*, antigen identified by monoclonal antibody Ki-67; *Nes*, nestin; *NeuroD1*, neurogenic differentiation 1; *Notch2*, notch 2; *Nrxn3*, neurexin 3; *Prom1*, prominin-1; *Prox1*, prospero homeobox 1; *Psa-Ncam*, polysialylated neural cell adhesion molecule; *Rpl32*, ribosomal protein L32; *S100b*, S100 protein, beta polypeptide, neural; *Slc1a2*, solute carrier family 1 (glial high affinity glutamate transporter), member 2; *Sox2*, SRY (sex determining region Y)-box 2; *Sox9*, SRY (sex determining region Y)-box 9; *Sp8*, trans-acting transcription factor 8; *Sp9*, trans-acting transcription factor 9; *Tbr2*, eomesodermin; *Tubb3*, tubulin, beta 3 class III.

Studies in the adult mouse brain demonstrated that NSCs are not homogenous cells, but rather a combination of distinct subpopulations recognizable mainly by their state of quiescence or activation. Neural stem cells display regional heterogeneity, which is acquired from their embryonic origin and niche patterning. Neural stem cells in the adult V-SZV niche originate from a subpopulation of embryonic radial glial cells, which became specified during development and maintain their quiescence until reactivation in adulthood [59]. Current progress in single-cell transcriptomics provides extremely useful information about the different states of NSCs and suggests a high degree of transcriptional dynamics throughout these states. Multiple molecular markers are currently used to distinguish particular NSC subsets, which in combination with the use of transgenic mice, flow cytometry, and single-cell RNA sequencing, reveal the complexity within the NSC population. Purification of V-SZV NSCs revealed four types of cells: dormant NSCs, quiescent NSCs (qNSCs), activated NSCs (aNSCs), and progenitor cells (NPCs). Most NSCs are qNSCs that express glial fibrillary acidic protein (GFAP) and prominin-1 (PROM1) markers. These cells give rise to activated, cycling and epidermal growth factor receptor (EGFR)-positive aNSCs, which differentiate into highly proliferative NPCs and finally to neuroblasts [60,61,62,63] (Figure 1).

Moreover, additional subpopulations in intermediate states have recently been discovered. Pseudotemporal ordering, based on single-cell transcription profiling data, revealed three subpopulations of aNSCs, which exhibit differential expression of specific genes, placing these subpopulations in a continuum between quiescence and activation [64]. In addition, single-cell RNA sequencing in dentate gyrus revealed that hippocampal NSCs also exhibit molecular heterogeneity [65].

**Figure 2:**
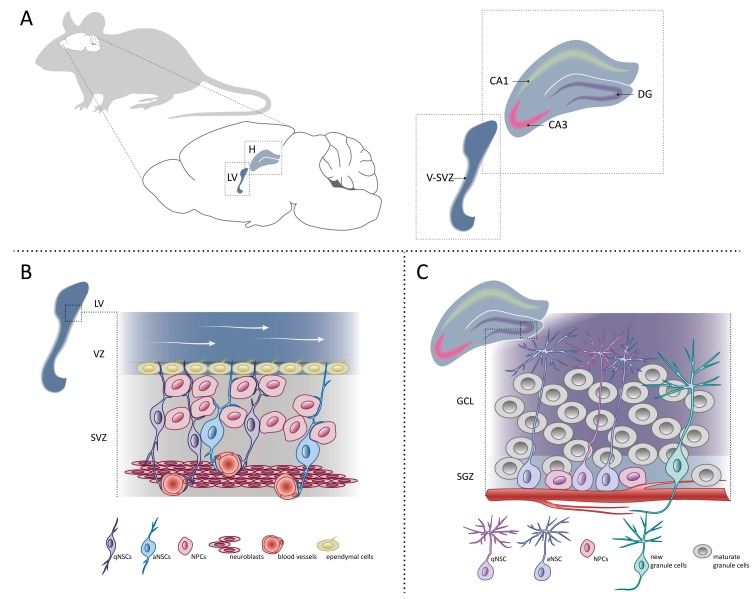
Neurogenesis in adult mouse brain. (**A**) Sagittal view of adult mouse brain focusing on two neurogenic niches where NSCs reside—the ventricular-subventricular zone (V-SVZ) of the lateral ventricle (LV) and dentate gyrus (DG) of the hippocampus (H). Cornu Ammonis 1 (CA1) and Cornu Ammonis 3 (CA3) subfields of the hippocampus are depicted. (**B**) Schematic representation of the organization and composition of the adult mouse V-SVZ niche. qNSCs share many characteristics with aNSCs, including contact with blood vessels. White arrows show the flow of the cerebrospinal fluid. (**C**) Schematic representation of the cell types present in the mouse subgranular zone (SGZ) and granule cell layer (GCL) in the dentate gyrus of the hippocampus.

The adult mouse brain contains two neurogenic niches located in V-SVZ and the dentate gyrus of the hippocampus. The neurogenic niche is a microenvironment supporting and nourishing NSCs through the secretion of local factors, nutrients and oxygen necessary for their maintenance. Local stimuli from the niche, as well as circulating blood factors can affect the NSC state and differentiation potential, and in consequence, neurogenesis in adult brain [66] (Figure 2).

Adult NSCs also receive feedback signals from cells at later stages in the lineage. For instance, neuroblasts secrete non-synaptic γ-aminobutyric acid (GABA) that binds to GABA type A receptor (GABA_A_R) expressed by qNSCs and inhibits their proliferation [67,68]. Interestingly, it was also shown that adult neurogenesis could be modulated depending on hunger or satiety, via hypothalamic control. The hypothalamus, a brain area regulating physiological states, provides long-range signals to the V-SZV niche and promotes proliferation of specific NSC populations [69].

## 4. Molecular Mechanism Underlying Brain Radiotherapy

The cytotoxicity caused by radiation is mainly the result of DNA damage. Radiation induces several forms of DNA damage, which include single-strand breaks, double-strand breaks, sugar and base modification, and DNA-protein crosslinking [70]. Among these, double-strand breaks are the dominant form of damage caused by ionizing radiation that when unrepaired can lead to lethality of cells [71]. In response to DNA damage, cell cycle checkpoints become activated to block cell cycle progression, allowing cells to repair the damage [72]. Depending on the phase of the cell cycle at which cells are damaged, the cells can be blocked at either the G_1_/S or G_2_/M checkpoints. If the damage is irreversible, apoptosis, programmed cell death, is triggered to eliminate the injured cells. Apoptosis after irradiation has been described in both neurogenic niches of experimental animals. Radiotherapy kills proliferating cells in V-SVZ of the brain in young adult rats [73]; similarly, apoptosis occurs in dentate gyrus of the adult rat hippocampus [74,75].

Radiation therapy reduces adult neurogenesis through two mechanisms. Ionizing radiation, by inducing acute apoptosis in dividing cells, reduces the pool of mitotic NSCs, mainly aNSCs and NPCs, and consequently reduces generation of new neurons [76,77]. However, at moderate doses of irradiation, proliferation in the V-SZV niche restarts 2–3 days after exposure by recruiting qNSCs [73,78]. Similar effects of irradiation on neurogenesis recovery have been reported in the hippocampus following moderate dose exposure [79]. A key feature of NSCs is their proliferative capacity that ensures regeneration of damaged tissue through the activation of qNSCs [61,78]. A vast majority of slowly dividing qNSCs survive a moderate dose of radiation exposure and enter the cell cycle to regenerate the irradiated neurogenic niche [78]. Transcriptomic analysis of qNSCs sorted from the V-SVZ zone of 2-month-old mice revealed that genes upregulated after whole-brain irradiation are mainly associated with cell cycle, DNA/RNA processes, translation, and ribosomal activity [80]. This illustrates the transcriptomic shift of irradiated qNSCs towards cell cycle entry. Interestingly, gene set enrichment analysis also showed enrichment in genes associated with the tricarboxylic acid cycle and respiratory electron transport, indicating that the cell cycle entry of qNSCs after radiation was accompanied by a shift toward an oxidative metabolism. Furthermore, it was shown that the GABA_A_R signaling regulates qNSC cell cycle entry by using specific GABA_A_R agonists/antagonists and that the radiation-induced depletion of neuroblasts, the major GABA source, provokes qNSCs to exit quiescence in the irradiated V-SVZ [78].

Radiation exposure of neonatal brain has been shown not only to diminish the cognitive function, but also to enhance carcinogenesis. The analysis shows that juvenile mouse V-SZV has a larger number of proliferating progenitors than the adult brain [81,82]. However, the neonatal progenitor cells have diminished ability to undergo proliferative arrest compared to adult progenitors and recover the proliferative capacity more rapidly. Thus, neuroblasts in neonates are derived from irradiated proliferating cells, and this may influence the level of genomic DNA alterations they contain and consequently their ability to become carcinogenic [81].

Another mechanism that affects neurogenesis after radiation exposure are changes within the NSC microenvironment. The exposure to high doses causes permanent inhibition of proliferation and neurogenesis in the neurogenic niche [83], which is a direct consequence of the changes in the NSC niche [84,85]. Even if qNSCs survive irradiation and, thus, are potentially able to reconstitute neurogenesis, such regeneration may be counteracted by sustained inflammation and vascular damage in the stem cell niche. Radiation may also lead to premature differentiation of neural precursors and adoption of glial fate [84,86,87]. After high doses of radiation, the neurogenic niche is chronically altered and generates a hostile environment. Experiments demonstrated that irradiated neuronal precursors are able to differentiate in vitro, but transplanted non-irradiated precursors cells are unable to differentiate in an irradiated hippocampus [84]. This illustrates that the alteration of neurogenesis that occurs following irradiation is largely due to modifications of the neurogenic niche. In irradiated mice, a marked increase in transforming growth factor β1 (TGF-β1) production by endothelial cells in the stem cell niche was observed. The increased synthesis of TGF-β1 by brain endothelial cells provokes qNSC dormancy and increases susceptibility of proliferative NSCs to apoptosis [85]. In co-cultures, irradiated brain endothelial cells induce apoptosis of NSCs via TGF-β/Smad3 signaling. Interestingly, the inhibition of TGF-β signaling improves neurogenesis in irradiated mice by preventing apoptosis of neural progenitors and by inducing proliferation of NSCs, and, consequently, restores production of new neurons [85].

Although radiation kills proliferating cells in both neurogenic niches, differential recovery of NSCs in V-SVZ and dentate gyrus of the hippocampus after moderate doses has been reported in the brains of young rats. While an initial response to radiation injury is similar in both neurogenic niches, the long-term effect on NSCs and neurogenesis in these two areas differs significantly. The dentate gyrus of the hippocampus is severely affected in the long term, whereas V-SVZ appears to recover with time [88].

Cranial irradiation not only affects the NSC populations, but also causes vascular damage. Irradiation disrupts the vasculature of the niche, reduces the microvessel area, the number of microvessels and the number of microvessel branching points in the hippocampus of young mice [89]. Proliferative neural precursor cells tend to be clustered around vessels [90]. This association is lost in the irradiated hippocampus, where the distance between microvessels and the resident NSC population is increased [84,91].

**Figure 3:**
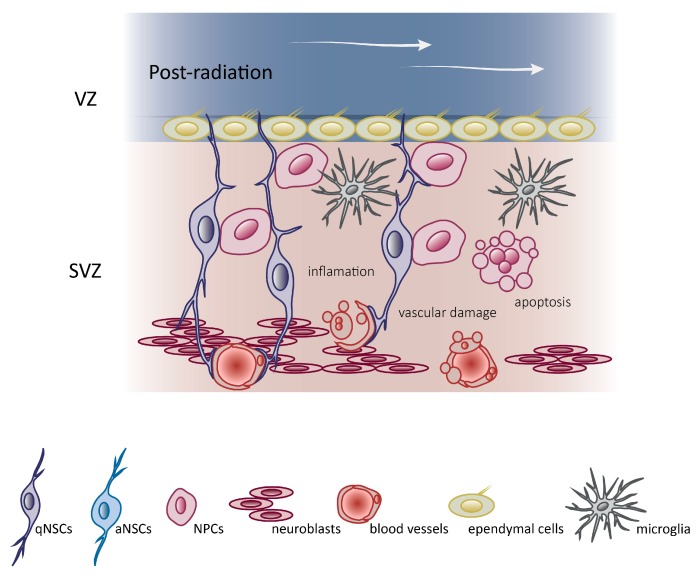
Radiation disrupts the V-SVZ niche. Schematic representation of mice V-SVZ niche after radiation. Following radiation, the V-SVZ niche shows a depletion of proliferating aNSCs, NPCs, and neuroblasts, a vascular damage and an increase in the number of microglia. Compare with schematic representation of mice V-SVZ niche in a pre-radiation condition (Figure 2B).

A microglial inflammatory response accompanied by an abnormal increase of cytokines occurs in NSC niches after brain radiation exposure and, in consequence, negatively affects neurogenesis and cognition. Microglia do not originate from NSCs, but differentiate through the monocyte lineage from hematopoietic stem cells and act as the resident macrophages of the central nervous system. Rola et al. [92] observed that after irradiation, reduced neurogenesis within the dentate gyrus of young mice occurs in conjunction with a chronic inflammatory reaction. An increase in the number of microglia present in the brain is correlated with increased radiation doses [93]. Whole-brain irradiation induces regionally specific pro-inflammatory environments with elevated expression of cytokines, including tumor necrosis factor α, interleukin 1 β and monocyte chemotactic protein 1 [94] (Figure 3).

## 5. Strategies to Minimize Radiation-Related Damages in the Neurogenic Niche

The high-precision technologies that individualize target volume and dose of radiation therapy are increasingly used to limit injury to neurogenic niches. These techniques have been described above; we mention for example SR that is increasingly used technique with mild toxicity to patients [95]. Similarly, PBT offers the potential to minimize late-onset damages [96]; maximal sparing of the healthy tissues also ensures the using of IMRT technology [29].

Much effort is currently dedicated to find pre-irradiation treatments that may prevent the negative effects of radiation on the niche and NSCs. This is particularly important in the course of irradiation of the juvenile brain, where the consequences are more severe in comparison to irradiation of the adult brain. Lithium was shown to reduce damage and enhance neurogenesis, and has been explored as a pre-treatment option. Pre-irradiation administration of lithium resulted in reduced apoptosis and microglial activation [97,98]. Lithium increases proliferation of hippocampal NSCs and rescues radiation-induced cell cycle arrest in vitro. Treatment with 3mM LiCl was sufficient to increase NSCs in S phase, boost neurosphere growth, and reduce DNA damage [99]. It was shown that much of the lithium effect in hippocampal progenitors is attributable to the activation of Wnt canonical pathway by inhibition of glycogen synthase kinase 3 [100]. Another neuroprotective agent that can be a useful supplement to hippocampal sparing is natural polyphenol resveratrol. Resveratrol was shown to inhibit radiation-induced apoptosis in the hippocampus [101] and has a neuroprotective effect on irradiated NSCs in hippocampal slice cultures [102]. The resveratrol’s neuroprotective effect was dependent on its ability to selectively induce expression of mitochondrial superoxide dismutase, enzyme whose function is to clear mitochondrial reactive oxygen species and, as a result, to reduce oxidative stress and damage [103]. Several studies demonstrated that melatonin, a regulator of circadian rhythm produced in the pineal gland, appeared to ameliorate radiation-induced injury in various organs of rats [104]. Melatonin is known to be an effective antioxidant that scavenges free radicals produced by radiation before they induce DNA damage, and it also stimulates activities of antioxidant enzymes [105]. It was shown that melatonin has a protective effect on NSCs against lipopolysaccharide-induced inflammation [106], decreases apoptosis, and upregulates neural stem cell marker nestin in the V-SZV zone of irradiated rats [107].

Neuroinflammation is a significant component of the brain’s response to radiation. Interleukin 6, a mediator of the inflammatory response produced by microglia, was found to block neuronal differentiation of hippocampal NSCs, and administering a common nonsteroidal anti-inflammatory drug indomethacin to irradiated rats partly restored neurogenesis [108]. Jenrow et al. [109] administered pro-inflammatory cytokine production inhibitor MW-151 following irradiation and demonstrated a treatment-induced increase in migratory neuroblasts within the dentate gyrus of the hippocampus of adult rats. The peroxisomal proliferator-activated receptors (PPARs) are ligand-activated transcription factors [110], which have been shown to confer neuroprotection in a variety of models [111]. Administration of PPARα agonist fenofibrate preserved hippocampal neurogenesis and prevented radiation-induced cognitive impairment [112,113]; the application of pioglitazone, the PPARγ agonist, significantly recovered cognitive impairment in irradiated rats [114]. Furthermore, radiation-induced impairment of hippocampal neurogenesis in rats was mitigated by using combined administration of avorvastatin and angiotensin converting enzyme inhibitor ramipril [115].

The effect of selective inhibition of autophagy on NSCs in the dentate gyrus after cerebral irradiation was studied using mice with neural-specific deletion of autophagy related 7 gene, which is involved in autophagy induction and autophagosome formation. Selective inhibition of autophagy reduced radiation-induced cell death and caspase-dependent apoptosis in the dentate gyrus and cerebellum; moreover, the levels of pro-inflammatory cytokines decreased [116]. These results suggest that autophagy might be another potential target for preventing radiotherapy-induced cell death and its associated long-term effects.

A new strategy that could be used to prevent radiation-induced injury is stem cell therapy. As cranial irradiation induces progressive depletion of NSCs, the use of NSCs replacement constitutes a novel alternative to combat radiation-induced cognitive decline. Studies demonstrated that irradiated rats engrafted with human NSCs (hNSCs) showed less decline in cognition when compared to irradiated animals. Transplantation promotes not only early, but also long-term recovery of the irradiated brain [117,118]. However, there are concerns regarding stem cell use due to the possibility of teratoma formation and immune rejection, which subsequently requires immunosuppression [119]. The hNSC-derived microvesicles then provide attractive alternatives to stem cells, avoiding teratoma formation in the brain and minimizing the host graft rejection. It was shown that cranial grafting of microvesicles secreted from hNSCs attenuates neuroinflammation and preserves the structural integrity of the irradiated microenviroment and consequently improves cognition of irradiated rats [120]. The supplementation of whole brain irradiated mice with fetal mouse NSCs, injected via the tail vein, led to exogenous NSCs differentiation into neuronal and glial lineages but moreover, NSCs also differentiated into brain endothelial cells, which was accompanied by the restoration of cerebral blood flow [121]. Radiation-related symptoms cannot be attributed only to the disruption of neurogenesis. Brain irradiation damages brain white matter and causes demyelination and oligodendrocytes have been investigated to be a target of high-dose radiation [122]. Oligodendrocyte progenitors, derived from human pluripotent stem cells and grafted to rat’s forebrain, were able to remyelinate the irradiated brain and to rescue animal’s cognitive deficits. Additional recovery from motor deficits requires concomitant oligodendrocyte progenitors transplantation into the cerebellum [123]. The effects of intranasal administration of human mesenchymal stem cells (hMSCs), as a neuroprotective strategy for cranial irradiation, was investigated by Soria et al. [124]. The transplantation of hMSCs alters the gene expression profile of irradiated brain, modulates genetic pathways associated with inflammation, immune system and cell motility, and reduces oxidative damage and neuronal loss in brains of irradiated mice. The authors demonstrated that intranasally delivered hMSCs promote radiation-induced brain injury repair and improved neurological function, and suggest the therapeutic use of hMSCs as a non-invasive approach to prevent neurological complications of radiotherapy.

Additional strategies that promise to support the treatment of brain tumors are metabolic therapies, such as caloric restriction, intermittent fasting or a ketogenic diet [125]. Current treatment of primary brain cancers utilizes a multifactorial approach involving maximal safe resection, followed by radiotherapy and simultaneous chemotherapy [126]. Under normal physiological conditions, brain cells obtain energy from either glucose or ketones. Tumor cells rely preferentially on anaerobic glycolysis rather than on respiration, a phenomenon known as the Warburg effect [127]. High glucose levels accelerate brain tumor growth and angiogenesis while preventing apoptosis [128]. A strong dependence on glucose renders cancer cells vulnerable to therapy that targets glucose metabolism. The restricted diet is, thus, well suited as a non-toxic metabolic therapy for the treatment of malignant brain cancers as demonstrated in many case reports [129,130,131]. Moreover, it has been demonstrated that chemotherapy and high-dose radiation, used in the treatment of brain tumors, creates a tumor microenviroment that is rich in glucose and glutamine and this can further contribute to tumor progression [132]. In the tumor microenviroment, the neoplastic cell populations are associated with macrophages/monocytes cells. These associated cell populations contribute to tumor progression through the release of pro-inflammatory and pro-angiogenic factors [132,133,134]. Nevertheless, several studies in rodents demonstrated that caloric restriction not only leads to reduced tumor growth but also mitigates inflammation, improves macrophages function [135], and lowers cytokines expression [136].

## 6. Concluding Remarks

Whether hippocampal neurogenesis persists throughout life in the human brain is not fully resolved. It was believed that the human hippocampus continues to generate new neurons, but a report by Sorrells et al. [53] concluded that neurogenesis does not continue in the human adult hippocampus, or is extremely rare. Moreover, this study also reminds us that simple translation of results from animal studies to humans may be problematic. On the contrary, persistent hippocampal neurogenesis was demonstrated in aging brains and detected in patients with mild cognitive impairments and Alzheimer’s disease [137,138]. Importantly, a study by Tobin et al. [137] also provided evidence that the extent of neurogenesis, particularly the number of newly forming neurons, is associated with better cognitive diagnosis. Nevertheless, they also showed that the number of neuroblasts greatly varied between individuals. The evidence for adult neurogenesis in the human brain comes from studies using thymidine analogs that are incorporated into the DNA of dividing cells, and from studies that only used immunohistochemistry to detect cell proliferation markers in human postmortem brains. It should be emphasized that there are many potential technical obstacle to studying post mortem brain tissues. One of them is the post mortem brain interval which can have deleterious effect on brain antigenicity and should be taken into consideration during tissue selection for analysis. Another important limitation that applies to studies of human neurogenesis are fixation time and tissue processing, the limitation of antibodies and marker specificity and interpersonal variability of marker expression. To finally resolve if neurogenesis persists in the human adult brain will need a more complete analysis by using for example, single-cell RNA sequencing, standardization of methodologies and the creation of an open-access brain bank from a large cohort of patients [139,140].

The human brain tumors classification is currently based mainly on microscopic morphology and immunochemistry; nevertheless, many tumors are characterized by a distinct molecular signature which enables their genomic classification. For instance, medulloblastomas comprise an explicit subgroup with distinct molecular characteristics [141,142] and provide a clear example of how a detailed understanding of genomics can guide the treatment procedure. Evidence that medulloblastomas, which display active Wnt-signaling pathway, lacks the blood–brain barrier and, therefore, are highly vulnerable to chemotherapy [143] led to a series of studies testing reduced-intensity radiotherapy in patients with this disease subtype [144]. This also shows that although recent technical advances in radiotherapy allow localized and concentrated treatment, reducing of radiotherapy for some types of brain tumors is one of the main challenges [144,145].

Although it is difficult to examine adult neurogenesis in humans, postnatal neurogenesis has been well studied in rodents. Animal studies have shown that proliferative and migratory capacities of neural precursors are disrupted by irradiation, however depletion of neuroblasts provokes qNSCs to exit quiescence and activate. The mechanisms that could explain reduction of neurogenesis and the resultant negative long-term side effects of radiation therapy, is the premature exhaustion of a finite NSC pool that is a detrimental consequence of aberrant NSCs activation [146] together with the chronic alteration of the neurogenic microenviroment. Nevertheless, radiotherapy is still the standard treatment strategy for most human brain tumors, which by increasing the radiation dose can lead to improved tumor outcomes. This ambiguity of radiation treatment is necessary to keep in mind when treating brain tumors. Determining the subcategories of individual tumors and following the expression of biomarkers in time will help in deciding which patients will benefit from radiation treatment.
